# Trophoblastic Tissue Reimplantation below the Spleen Following Laparoscopic Bilateral Salpingectomy for Ectopic Tubal Pregnancy: A Case Report

**DOI:** 10.3390/medicina59040701

**Published:** 2023-04-03

**Authors:** Dominyka Surgontaitė, Artūras Sukovas, Dalia Regina Railaitė, Tautvydas Jankauskas, Arnoldas Bartusevičius, Eglė Bartusevičienė

**Affiliations:** 1 Department of Obstetrics and Gynaecology, Lithuanian University of Health Sciences, 50161 Kaunas, Lithuania; 2 Department of Interventional Radiology, Lithuanian University of Health Sciences, 50161 Kaunas, Lithuania

**Keywords:** trophoblastic tissue reimplantation, embolization, methotrexate

## Abstract

Background: Trophoblastic tissue reimplantation after laparoscopic salpingectomy is a very rare complication. These cases may present a diagnostic challenge and the majority of patients need a surgical treatment. Case presentation: A 31-year-old patient came to a tertiary referral center for nausea and pain in the upper left abdominal quadrant. Ultrasound and abdominal CT scan showed a 68 × 60 × 87 mm size heterogenic mass below the spleen with arterial extravasation from the lower spleen pole. Recent history of surgery for ectopic pregnancy and serum hCG testing allowed to diagnose extratubal secondary trophoblastic tissue reimplantation below the spleen. Embolization of the bleeding vessel and successful treatment with methotrexate was achieved. Conclusions: In cases of a nondisseminated trophoblastic tissue reimplantation, consider embolization and treatment with methotrexate if the patient is hemodynamically stable; thus, secondary surgical treatment is preventable.

## 1. Introduction

Ectopic pregnancy occurs in 1–2% of all pregnancies [1,2]. The rate of persistent ectopic pregnancy after expectant management, surgical or methotrexate treatment is reported in 4–15% of the cases [3,4]. Ectopic pregnancy tissue reimplantation is a very rare complication. A total of 25 cases of reimplantation of trophoblastic tissue following laparoscopic removal of ectopic pregnancy have been reported in the period from January 1989 to January 2018, and only a few of these cases describe peritoneal trophoblastic implantation following laparoscopic salpingectomy [5,6,7,8,9]. To our knowledge, only two similar cases have been reported since 2019 [10,11].

In the latest ectopic pregnancy treatment guidelines, there is no necessary follow-up after salpingectomy, as it is considered a definitive treatment for tubal pregnancy [3]. In this report, we present a clinical case of trophoblastic tissue reimplantation below the spleen following laparoscopic bilateral salpingectomy.

## 2. Clinical Case

A 31-year-old woman was sent to the emergency department of a tertiary referral center due to an unknown diagnosis. The patient was feeling nauseous and complained of intensifying pain in the upper left abdominal quadrant.

The woman was hemodynamically stable. During physical examination, abdominal muscle tension was observed, and there was no rebound tenderness. Ultrasound revealed a mass in the upper left side of the abdomen and a small amount of unclear fluid around the intestines. In the blood test, only leukocytosis of 17.3 × 10^9^/L was abnormal.

Three weeks earlier, in a regional hospital, she was diagnosed with ectopic pregnancy (7 weeks of gestation) in her left fallopian tube. Left laparoscopic salpingostomy was planned, although salpingectomy was performed due to heavy bleeding. Data on ultrasound findings before surgery was not available. The serum human chorionic gonadotropin (hCG) concentration was not tested. Histological reports showed no chorionic villus, and only several trophoblasts were seen in the surgically removed material. In the past, the woman had had two normal deliveries, pelvic inflammatory disease and one more ectopic pregnancy in her right fallopian tube. Therefore, she underwent laparoscopic right salpingectomy.

**Figure 1:**
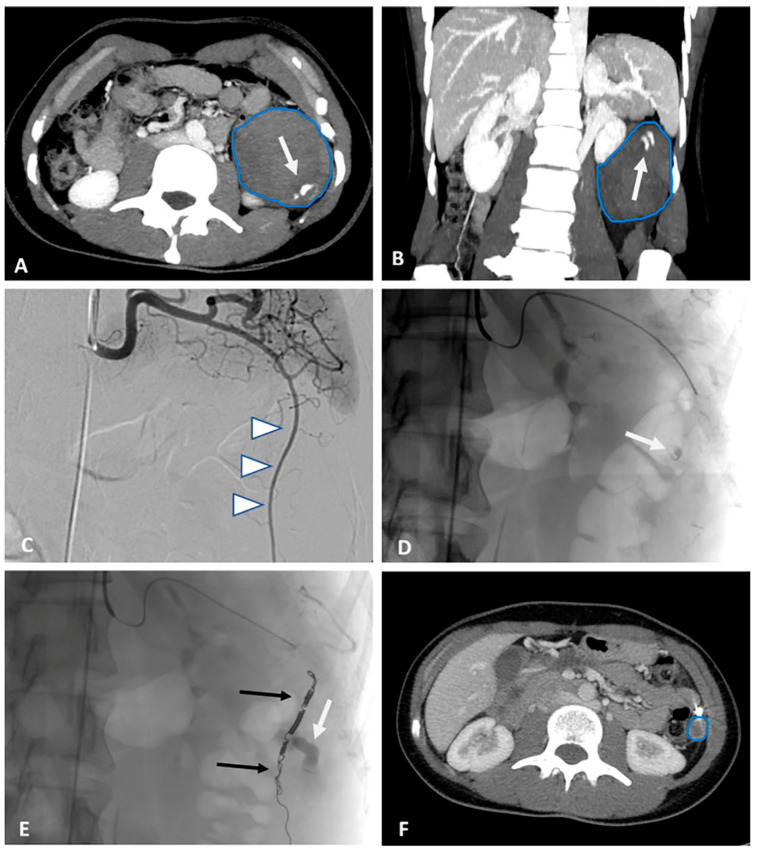
Axial and coronal 15 mm maximum intensity projections (contrast-enhanced computed tomography (CE-CT) in the emergency department, prior to embolization), large hematoma is seen in the upper left abdomen (blue outline) with active arterial extravasation (white arrow) (**A**,**B**). Digital subtraction angiography of coeliac trunk shows unusual large arterial branch (white arrowheads) descending from the splenic artery to lower abdomen—most likely hypertrophied left epiploic artery (**C**). Selective angiography with micro catheter shows slow arterial extravasation (white arrow) (**D**). Post-embolization image (**E**) with embolizing coils (black arrows) above and below extravasation site, also stagnated contrast medium in hematoma can be seen (white arrow). Significant size reduction of hematoma is seen at 7 month follow-up CT image (blue outline) (**F**).

**Chart 1:**
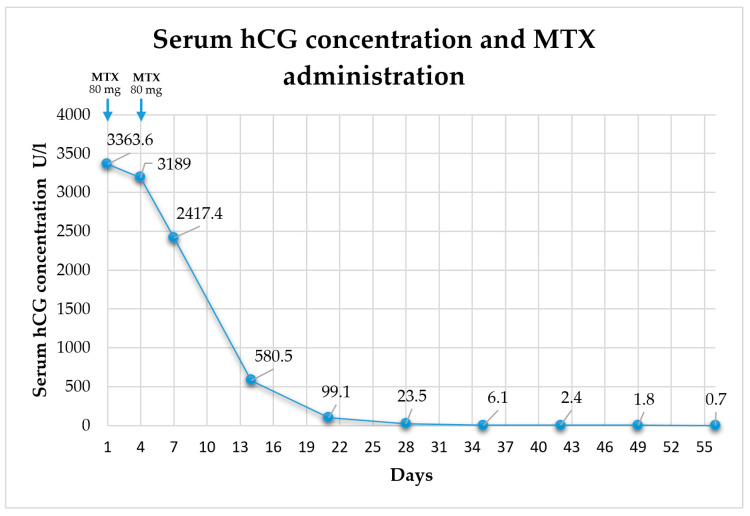
Serum hCG concentration drop and methotrexate administration.

A computed tomography (CT) scan was performed to differentiate the mass found on ultrasound. Abdominal and pelvic CT showed a 68 × 60 × 87 mm heterogenic mass, likely a hematoma, which was below the spleen. Pelvis was filled with hemorrhagic fluid, and the uterus was enlarged. Arterial extravasation was spotted from the lower spleen pole. Conventional angiography of the splenic artery was performed, and extravasation was identified in the region below the spleen. The vessel was embolized (Figure 1A–E). Due to the unknown origin of the hematoma and the recent history of surgery for ectopic pregnancy, and gynecologist consultation was sought. The gynecologist ordered a urine pregnancy test, which was positive. The serum human chorionic gonadotropin (hCG) concentration was 3363.6 U/L. The clinical case was considered a rare pathology of extratubal secondary trophoblastic tissue reimplantation. As the bleeding was stopped with embolization, a conservative two-dose methotrexate (MTX) treatment protocol with MTX 50 mg per square meter of body surface area intramuscularly (mg/m^2^ BSA i/m) on day 1 and day 4 was administered. Abdominal pain did not intensify; subjectively, the woman started feeling better. The decrease in serum hCG concentration between days 4 and 7 was 24.1% (i.e., more than 15%); thus, hCG testing was repeated weekly (Chart 1). Menstruation renewed 29 days after the first dose of MTX. Seven months later, the CT scan showed that the size of the heterogenic mass below the spleen decreased to 18 × 14 × 16 mm (Figure 1F).

## 3. Discussion

The extratubal secondary trophoblastic tissue reimplantation following surgical removal of an ectopic pregnancy is estimated to be found in 1–1.9% of the cases, and it seems that there is an equal distribution between salpingostomy and salpingectomy [9]. Given that these cases may present a diagnostic challenge and are usually detected via sudden abdominal pain and acute hemoperitoneum, it is necessary to prevent and diagnose such cases in the early period after the primary surgery [10]. Current guidelines state that hCG follow-up after laparoscopic salpingostomy should be made 7 days after the surgery, then one measurement per week until a negative result is obtained. A subsequent urine pregnancy test could be performed 3 weeks after salpingectomy [2,3].

In our clinical case, salpingostomy was considered first, because the patient previously had an ectopic pregnancy and underwent contralateral laparoscopic salpingectomy. When the bleeding began, salpingectomy was performed, and there was a possible dissemination of trophoblastic tissue. Our case supports the recommendation of some authors regarding postoperative hCG monitoring if the specimen was fragmented during salpingectomy and if there could have been spillage of the trophoblastic tissue [11,12]. It would have been reasonable to have performed serial hCG measurements given the attempted salpingostomy. In addition, it is important to avoid iatrogenic dissemination of trophoblast tissue through surgery. It is recommended to limit the use of the Trendelenburg position thorough suction and the removal of blood clots, abundant irrigation of the abdominal cavity, and careful removal of the pregnancy tissue [5,9].

To diagnose the peritoneal reimplantation of trophoblastic tissue can be very difficult. The symptoms vary from abdominal pain and vaginal bleeding to hemorrhagic shock. The time period from the first operation until the patient returned to the hospital varied from 7 to 51 days. Therefore, it is important to consider the possibility of persistent or reimplanted trophoblastic tissue even though weeks have passed since the primary operation [9,11]. In our case, the patient was hospitalized 21 days after the surgery for ectopic pregnancy due to abdominal pain and intra-abdominal bleeding. These cases are hard to interpret through ultrasound, and often auxiliary examinations (abdominal, pelvic CT and/or magnetic resonance imaging (MRI)) are requested. MRI could be the key diagnostic method if hCG continues to rise. It also assists in determining the location of the possible reimplantation and helps to plan surgical steps [13,14]. In the presented case, trophoblastic tissue reimplantation diagnosis was not easily made. The patient was thoroughly investigated by an emergency physician and a surgeon. After a CT scan, hemorrhage of an unknown origin was considered; accordingly, the bleeding was managed with embolization. A certain diagnosis of extratubal secondary trophoblastic tissue reimplantation was made only after a gynecologist’s consultation, urine pregnancy test, and serum hCG testing.

The treatment of trophoblastic tissue reimplantation depends on whether the patient is hemodynamically stable [8,9]. Schyum AC et al. presented a literature review of trophoblastic tissue reimplantation in which all patients needed surgical treatment: laparoscopy in 40% (10/25) and laparotomy in 28% (7/25). The surgical method was not stated in eight of the twenty-five cases. MTX was only used as an additional treatment during or after the second operation [9]. However, MTX can be one of the primary treatment options for hemodynamically stable patients [10]. Our clinical case suggests that if a nondisseminated trophoblastic tissue implant after salpingectomy is diagnosed in a hemodynamically stable patient, embolization of the bleeding vessel and treatment with MTX can be considered. After embolization, MTX concentrations in trophoblastic tissue were appropriate as the serum hCG count continued to fall.

## 4. Conclusions

The diagnosis of the peritoneal reimplantation of trophoblastic tissue can be very challenging; thus, it is very important to avoid iatrogenic dissemination of trophoblast tissue through operation and to follow up serum hCG even after salpingectomy, especially when the conditions of trophoblastic tissue removal are questionable. If a nondisseminated trophoblastic tissue implant after salpingectomy is diagnosed prior to the development of massive intra-abdominal hemorrhage, we suggest considering embolization of the bleeding vessel and treatment with methotrexate.

## Data Availability

Not applicable.
